# Inadequate dietary diversity practices and associated factors among postpartum mothers in Gambella town, Southwest Ethiopia

**DOI:** 10.1038/s41598-023-29962-6

**Published:** 2023-05-04

**Authors:** Taye Teferi, Genet Endalk, Girum Meseret Ayenew, Netsanet Fentahun

**Affiliations:** 1 Department of Nutrition and Dietetics, School of Public Health, College of Medicine and Health Science, Bahir Dar University, Bahir Dar, Ethiopia; 2 Amhara Public Health Institute, Bahir Dar, Ethiopia

**Keywords:** Medical research, Epidemiology

## Abstract

The shortage of diversified diets in lactating postpartum mothers is a severe problem in developing countries. The promotion of diverse diets is important to improving micronutrient nourishment and adequate energy intake for lactating mothers. To date, there is limited evidence regarding inadequate dietary diversity practices among lactating postpartum mothers in Gambella region. The study is aimed to determine inadequate dietary diversity practice and associated factors among lactating postpartum mothers in Gambella city, southwest Ethiopia. Mixed methods were employed on 407 randomly selected lactating postpartum mothers and 15 purposively selected key informants from February 28 to March 24, 2021. A pre-tested questionnaire and interview guide were used for data collection. Data were analyzed using Statistical Package for the Social Sciences version 21 software. Binary logistic regression models were used to determine the associated factors of dietary diversity. Qualitative data were analyzed manually through a thematic approach. The prevalence of inadequate dietary diversity practice was 60.2%. Having no education (AOR = 3.74, 95% CI: 1.18, 11.88), employed women(AOR = 0.37, 95% CI: 0.18, 0.75), meal frequency < 3 meals (AOR = 2.92, 95% CI: 1.04, 8.71), time taken to market > 30 min (AOR = 4.20, 95% CI: 2.01, 8.76), not received nutrition education (AOR = 2.0, 95% CI:1.09, 3.68), having home gardening (AOR = 0.32, 95% CI: 0.18, 0.57) and having big animals (AOR = 0.12, 95% CI: 0.05, 0.29) were significant factors of inadequate dietary diversity practice. Diet habits, food taboos, low social status of women in ownership of household assets, low family support, order of feeding, child preference for resource distribution in a polygamous family, and health care provider’s advice were the main mentioned reasons for inadequate dietary diversity practices. The prevalence of inadequate dietary diversity practices were high compared to previous studies. Having no education, employed women, meal frequency < 3 meals, time taken to market > 30 min, not receiving nutrition education, having a home garden, and having big animals were significant factors of inadequate dietary diversity practice. Nutrition intervention focused on nutrition education to increase meal frequency should be provided for lactating postpartum mothers to improve inadequate dietary diversity practices.

## Introduction

Maternal nutrition and dietary practice are essential public health problems in developing countries particularly in Africa. Lactating mothers are vulnerable to malnutrition due to sub-optimal feeding practices, lacto-genesis process, and increased nutrient needs during lactation^1^. Promotion of diverse diets is one of cost effective approach to improving macronutrient and micronutrient deficiencies for lactating women^2^. Consumption of diverse diets delivers different essential nutrients to body normal growth as well as prevention of any disease^3^.

Improved dietary diversity during the postpartum period are crucial for immediate and long term maternal and child health outcomes. During pregnancy and lactation, nutritional demand gets higher than ever^4^. This increased physiological demand needs high dietary diversity during postpartum period^5^. However, the majority of lactating women in developing countries have inadequate diversity practice and nutrient intakes^6–9^.

Post-partum lactating mothers’ malnutrition is the major problem in Ethiopia. Food restrictions are the common practices in most part of the country^10,11^. Evidence on maternal dietary diversity practice during lactation is essential to achieve the 2025 global nutrition target and reduce maternal and child mortalities. In the highlands of Ethiopia, evidence about dietary diversity practice and its determinants among postpartum lactating mothers are available^7,12,13^. However, there is limited evidence in lowlands of the country like Gambella City. Therefore, this study is aimed to determine inadequate dietary diversity practice and associated factors among lactating postpartum mothers in Gambella City, southwest Ethiopia.

## Methods

### Study design and setting

An institutional based mixed cross sectional study design was employed from February 28 to March 30, 2021 at two public hospitals, Gambella city. The Gambella Peoples National Regional State is located in the Southwestern part of Ethiopia about 753 km west of Addis Ababa perched at an elevation of 526 m above sea level. Gambella city has an estimated population of 63,220 of which 50.8% (32,116) are females and the rest 49.2% (31,104) are males.

Gambella city has Gambella General Hospital (GGH) and Gambella City Primary Hospital (GTPH). These both hospitals have catchment population of more than 450,000 and 63,220 people respectively. Currently both hospitals are providing maternal and child health care services such as antenatal care (ANC), expanded program on immunization (EPI) and postnatal care (PNC) services^14^. The hospitals had service coverage of 68% and 90% early PNC and EPI respectively in GCPH and 63% and 92% early PNC and EPI respectively in GGH^15^.

### Study population

The source population was all lactating post-partum mothers in Gambella City. Study populations were lactating post-partum mothers who visited the selected health facilities for PNC and EPI service during the specified data collection period for quantitative section and key informants such as lactating post-partum mothers, health workers, health extension workers, community leaders and religious leaders for qualitative section. Lactating postpartum mothers and being resident of Gambella city for at least 6 months were included.

### Sample size determination and sampling procedure

#### For quantitative data

The sample size was determined using single population proportion formula with the following assumptions: the proportion of dietary diversity practice 56.4%^16^, 95% of the confidence interval, and 5% marginal error. By adding a 10% non-response rate, the final sample size was 416.

First the number of lactating postpartum women attended EPI and PNC services per month in each hospital were estimated. The estimated number of lactating postpartum mothers who came for PNC and EPI services in each hospital in average was 374 and 320 in GGH and GCPH respectively. Sample size was proportionally allocated for both health facilities providing PNC and EPI service based on the total population they serve. After proportional allocation employed, then, the Kth interval was calculated and that was 2 for both hospitals. The initial study participant was the first client who attended PNC or EPI Service during first day of data collection period, then systematic random sampling technique was employed every k = 2 interval for both health facilities until the required number was reached.

#### For qualitative data

The qualitative sample was consisted of purposively selected 3 health extension workers, 2 health professionals who provide maternal health services, 2 community leaders, 2 religious leaders and 6 lactating post-partum mothers.

### Data collection procedure and measurements

*Minimum Women Dietary Diversity Score (MWDDS)* was sum of consumed food groups within 24 h by using 10 food groups and defined adequate DDS if they consume more than or equal to 5 food group within 24 h^17–19^.

*Food security* was defined as a state in which all people at all times have both physical and economic access to sufficient food to meet their dietary needs for a productive and healthy life. It was measured using HFIAS Guideline^20^.

Pretested structured questionnaire was used to collect the data. Socio-demographic, socio-cultural, meal frequency, health service usage practice, food groups, HFIAS, market access, crop and livestock diversity questions were included in the questionnaire. Two clinical nurses and one diploma nurse and principal investigator were recruited as data collectors and supervisor’s respectively. The in–depth interviews were made using semi structured guide and was conducted at hospitals. Each interview was tape-recorded and transcribed on the same day of the interview sessions.

### Data quality assurance

The quality of data was assured at the maximum attainable level by using standardized adapted questionnaire and following the necessary procedures in order to get the intended results. Pre-test was carried out at Itang health center on 5% sample size. Some modification was made on the data collection tools based on the gap identified during pretest interview to enhance reliability. Data collectors and supervisor were received two-day training on the purpose and procedure of data collection by principal investigator. The training session was addressed sensitive questions. Furthermore, the questionnaires were checked for completeness and correctness on daily basis by immediate supervisors.

To ensure trustworthiness data were collected from many participants and triangulated. Findings of the data analysis were brought back to the original participants to seek their input concerning the accuracy, completeness and interpretation of data.

### Data management and statistical analysis

The data was cleaned and coded before data entry and entered into EPI-DATA version 3.1 and then exported to SPSS version 21 for cleaning and analysis. Binary logistic regression model was used to identify independent predictors of dietary diversity practice. Variables having *p*-value < 0.25 in the bivariable analysis were further entered into the multivariable logistic regression model. Variables having *P*-value < 0.05 in the multivariable logistic regression analysis were considered as independent predictors of dietary diversity practice. The Hosmer–Lemeshow goodness of- fit test used to assess the fitness of the model.

Thematic analysis framework was used to analyze the qualitative data. The main themes in the data were selected by writing short phrases, ideas and concepts. Themes were achieved by reading the transcripts in several times and grouping. Interpretation of findings were done through data analyzed by data reduction which was achieved by comparing and contrasting data and cutting and pasting similar quotes together and the individual quotes. The relationship between the quotes, and the links between the data as a whole were seen.

### Ethical issues

Ethical clearance was obtained from Institutional Review Board, of College of Medicine and Health Sciences, Bahir Dar University. All methods were performed in accordance with the relevant guidelines and regulations. Official support letter was also obtained from Gambella regional state health Bureau to both hospitals. Hospitals’ senior management team was briefed on the objectives of the study and permission to conduct the study was obtained from the hospitals’ medical director. Informed consent was asked obtained from the study participants to confirm willingness for participation after explaining the objective of the study. The information provided by each respondent was kept confidential through anonymous recording and coding of questionnaire.

## Results

### Socio-demographic characteristics

A total of 407 respondents with a response rate of 97.8% participated in the study. The mean age of respondents was 28.67(+ 5.79 SD). More than half (55.5%) of participants' occupations were housewives. Two hundred eighty-eight (70.8%) mothers had a family size of 4 to 6 (Table 1).

**Table 1:** Socio-demographic characteristics of study participants among lactating postpartum mothers in Gambella city (n = 407), 2021.

Variables	Category	Frequency	Percent
Age	18–24	101	24.8
	25–29	154	37.8
	30–39	130	31.9
	40–49	22	5.4
Religion	Protestant	312	76.7
	Orthodox	44	10.8
	Muslim	37	9.1
	Catholic	8	2.0
	Adventist	6	1.5
Ethnicity	Nuer	145	35.6
	Agnuak	118	29.0
	Oromo	56	13.8
	Amhara	34	8.4
	Tigrie	19	4.7
Marital status	Single	6	1.5
	Married	381	93.6
	Divorced	17	4.2
	Widowed	3	
Educational status	No education	47	11.5
	Primary	181	44.5
	Secondary	111	27.3
	More than secondary	68	16.7
Occupation	House wife	226	55.5
	Employee	99	24.57
	Merchant	66	16.2
	Daily laborer	16	3.9
Monthly income (ETB)	≤ 3500	109	26.8
	3501–4500	116	28.5
	4501–7200	90	22.1
	> 7200	92	22.6
Family size	1–3	101	24.8
	4–6	288	70.8
	Above 6	18	4.4

*Others: silite, Kefa and Wolaita.

### Health service and socio-cultural characteristics

About 92% (372) and 88% (359) 88 mothers had ANC and PNC follow up respectively. A total of 287 (70.5%) of them got nutrition education during their follow-up. Ninety-four (23.1%) study participants had a polygamous relationship. About 48 (11.8%) of the study participants were married before the age of 18 years old. One hundred eighty-three (45%) study participants have experienced a shortage of time to prepare food due to workload (Table 2).

**Table 2:** Health, nutrition service and socio-cultural characteristics of study participants among lactating postpartum mothers in Gambella city (n = 407), 2021.

Variables	Category	Frequency	Percent
Have ANC follow Up	Yes	372	91.4
	No	35	8.6
Frequency of ANC follow up	No	35	8.6
	1–2	82	20.15
	3–4	285	70.02
	More than 4	4	1.0
PNC follow up	Yes	359	88.2
	No	48	11.8
Nutrition education	Yes	287	70.5
	No	120	29.5
Polygamy	Yes	94	23.1
	No	313	76.9
Age at marriage	< 18 years	48	11.8
	≥ 18 years	359	88.2
Time shortage to prepare food due to workload	Yes	183	45
	No	224	55

### Market access, crop and livestock diversity

A total of 336 (82.2%) participants have lived nearby the market. About 315 (77.4%) participants spent time to market less than 30 min. Regarding the food exchange of participants, 373 (91.6%) of them stated that they didn’t exchange their food products in the market. From 407 study participants, 121 (29.7%) were reported they had a home garden and the majority 367 (90.2%) of participants did not produce pulses. About 162 (39.8%) of study participants had small animals. Only 55 (13.5%) of study participants had big animals (Table 3).

**Table 3:** Market access, crop and livestock diversity among lactating postpartum mothers in Gambella town southwest Ethiopia, 2021.

Variables	Category	Frequency	Percent
Distance of market ≤ 1 km	Yes	336	82.6
	No	71	17.4
Time take to market ≤ 30 min	Yes	315	77.4
	No	92	22.6
Exchange of food	Yes	34	8.4
	No	373	91.6
Home garden	Yes	121	29.7
	No	286	70.3
Produce pulse	Yes	40	9.8
	No	367	90.2
Have small animals	Yes	162	39.8
	No	245	60.2
Have big animals	Yes	55	13.5
	No	352	86.5

### Dietary diversity practice among lactating postpartum women

**Figure 1:**
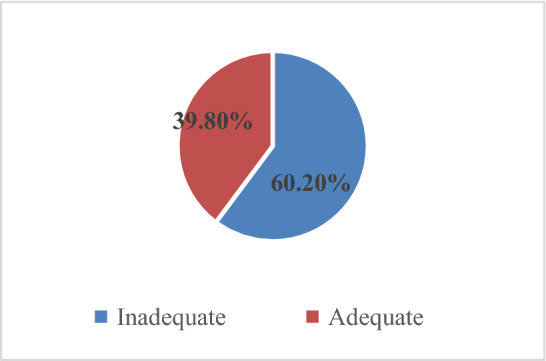
The prevalence of inadequate dietary diversity practice among lactating postpartum mothers in Gambella City, Southwest Ethiopia, 2021.

The prevalence of inadequate dietary diversity practice among lactating postpartum mothers was 60. 2% during the preceding 24 h of the survey. (Fig. 1) The majority of the study participants consume 3–4 food groups (56.8%) and 34.6% of the respondents consumed 5 food groups. Most of the respondents consumed all starchy staples 402 (98.8%) and 349 (85.5%) of respondents consumed other vegetables such as onion and tomatoes. About 216 (53.1%) of respondents consumed Vitamin A rich dark green leafy vegetables. Only 96 (23.6%) of study participants were consuming other Vitamin A-rich fruits and vegetables. Nuts and seeds were consumed by only 39 (9.6%) of respondents. Other fruits like lemon and apple are less consumed 9 (2.2%) by study participants. A total of 114 (28%) of the lactating mothers were food insecure. The mean meal frequency of lactating mothers was 3.26. A total of 234 (57.5%) study participants have consumed 3 meals per day within the past 7 days.

### Factors associated with dietary diversity practice

Mother’s educational status, occupation of mother, polygamy, age of marriage, shortage of time to prepare food, meal frequency, exchange of food, time take to market, ANC and, PNC follow up, nutrition education during follow up, home garden practice, pulse production, having small animals, having big animals, and household food insecurity had *P*-value less than 0.25 at bivariable logistic regression.

In multiple logistic regression analyses, mothers’ educational status, occupation of mothers, meal frequency, time taken to market, nutrition education during follow-up, having a home garden, and having big animals were associated factors with inadequate dietary diversity practice of lactating postpartum mothers.

Mothers who had no education were 3.74 times more likely to have inadequate dietary diversity practice compared to mothers who complete more than secondary education (AOR = 3.74,95% CI: 1.18, 11.88). Regarding the occupation of mothers, mothers who were employed were 63% times less likely to have inadequate dietary diversity practice compared to mothers who were housewives (AOR = 0.37, 95% CI: 0.18, 0.75).

Mothers who had less than three meals per day were 2.92 times more likely to have inadequate dietary diversity practice compared to mothers who had more than three meals per day (AOR = 2.92, 95% CI: 1.04, 8.17). Time taken to market, mothers who spent more than 30 min to market were 4.2 times more likely to have inadequate dietary diversity practice compared to mothers who spent less than or equal to 30 min to market (AOR = 4.20, 95% CI: 2.01, 8.76).

Nutrition education of lactating postpartum mothers during ANC and PNC follow up, mothers who didn’t get nutrition education during ANC and PNC follow up were 2 times more likely to have inadequate dietary diversity practice compared to their counterpart (AOR = 2.0, 95% CI: 1.09, 3.68).

Mothers who had home gardens were 68% times less likely to have inadequate dietary diversity practice compared to those who hadn’t home gardens (AOR = 0.32, 95% CI: 0.18, 0.57). Also, mothers who had big animals were 88% times less likely to have inadequate dietary diversity practice compared to their counterparts (AOR = 0.12, 95% CI: 0.05, 0.29) (Table 4).

**Table 4:** Bivariable and multivariable associations of factors with dietary diversity practice among lactating postpartum mothers in Gambella town, Gambella region, southwest Ethiopia, 2021.

Variables		Inadequate DDS	Adequate DDS	COR (95% CI)	AOR (95% CI)
Education	No education	37	10	5.29 (2.26, 12.36)	3.74 (1.18, 11.88)
	Primary	122	59	2.95 (1.66, 5.25)	2.52 (1.06, 5.99)
	Secondary	58	53	1.56 (0.85, 2.88)	1.50 (0.63, 3.55)
	More than secondary	28	40	1	1
Occupation	House wife	152	74	1	1
	Employed	39	61	0.32 (0.19, 0.52)	0.37 (0.18, 0.75)
	Merchant	41	24	0.80 (0.45, 1.41)	0.42 (0.20, 0.89)
	Daily laborer	13	3	2.11 (0.58, 7.63)	1.15 (0.19, 6.92)
	> 7200	39	53	1	
Have co-wives	Yes	67	27	1.88 (1.14, 3.10)	1.99 (0.99, 4.02)
	No	178	135	1	1
Shortage of time to prepare food	Yes	128	55	2.13 (1.41, 3.21)	1.56 (0.94, 2.61)
	No	117	107	1	
Meal frequency	Less than 3 meals	25	10	4.27 (1.90, 9.60)	2.92 (1.04, 8.17) **
	3 meals	169	65	4.43 (2.83, 6.95)	4.24 (2.47, 7.28) *
	> 3 meals	51	87	1	1
Time takes to market	≤ 30 min	173	142	1	
	More than 30 min	72	20	2.96 (1.72, 5.09) *	4.20 (2.01, 8.76) **
Have nutrition education	Yes	154	133	1	
	No	91	29	2.71 (1.68, 4.37) *	2.00 (1.09, 3.68) **
Home garden	Yes	50	71	0.33 (0.21, 0.51) *	0.32(0.18, 0.57) **
	No	195	91	1	
Have big animals	Yes	13	42	0.16 (0.08, 0.31) *	0.12 (0.05, 0.29) **
	No	232	120	1	1

*P < 0.25 during bivariable analysis; **P < 0.05 during multivariable analysis.

### Barriers of dietary diversity practice: qualitative finding

Frequently mentioned reasons for poor dietary diversity practice from qualitative participants were food practice, cultural practice, and health care practice.

#### Food practices

##### Diet habit

Almost all participants said that lactating mothers consumed the same staple food at breakfast, lunch, and dinner. They mentioned women have limited income and accessibility to obtain a more varied meal pattern were main reason consumed same staple food.

##### Food avoidance/food taboos

The other barrier that hider dietary diversity practice of mothers was food avoidance and food taboo during lactation. Most key informants underlined that eating some types of food can affect the health of either the mother or the baby. Almost all key informants reported that solid foods like “kollo”, “dry fish meat” and “bread” are taboo during lactation.

#### Cultural practice

##### Low social status of women in ownership of household assets

The other qualitative finding that hinders dietary diversity practice of lactating mothers was the low social status of women on ownership of household assets like chattels. Most of the key informants reported that culturally it is husbands' right to decide on the use and sale of essential family assets like cows.

##### Low family support

Low family support to lactating mothers during the postpartum period is a challenge that aggravates inadequate dietary diversity practice. Most participants mentioned that the preparation of food for the household members is relaying on mothers.

##### Order of feeding

Differences in social status and the hierarchy of men and women when referring to the order of eating at mealtime is an obstacle to having adequate dietary diversity of mothers. The majority of informants reported that culturally it is the habit of the woman to eat after all family members have eaten. There is some inconsistency towards the order of feeding, some key informants gave answer lactating postpartum mothers eat their food prior to other family members. Eating lately can attribute to having lactating women gain a small portion of food and items which leads to having inadequate dietary diversity.

##### Child preference for resource distribution in a polygamous family

The other sociocultural barrier that can hinder the dietary diversity of lactating postpartum mothers is related to polygamy. Many respondents stated that the Husband shares his money among his wives if he has many wives. Some respondents argued that husbands share more amount of money with the wife who had female children. Some stated that money is divided equally to all wives.

#### Health care practice

##### Health care providers’ advice

The other sociocultural barrier which hinders the dietary diversity of lactating mothers is the health care provider’s advice. Lactating mothers trust both traditional healers and professional health care providers. Some misinformation given by traditional healers can affect the nutritional status and health of mothers. Some respondents avoid some food items from their diet because their doctor is not informing them these food items have no side effects.

## Discussion

The aim of this study was to assess inadequate dietary diversity practices and associated factors among lactating postpartum mothers. The prevalence of the inadequate dietary diversity practices was 60.2%. This finding lower than the FAO guideline recommendation that all of the mothers should achieve the minimum dietary diversity scores to have adequate micronutrient adequacy^1^.

In this study, the prevalence of the inadequate dietary diversity practices is higher than studies done in Debere Tabor, Amhara, Dire Dawa and Angecha district southern nation nationalities region^21–23^ and in urban Nepal and Nigeria^24,25^. The possible reasons might be difference in food security status, occupational status, socio-cultural and nutrition literacy.

The magnitude of inadequate dietary diversity practices in this study is lower than a survey conducted in Finote Selam town^26^. This might be due to nutrition education provided to lactating mothers during ANC and PNC follow up. Small proportion of the study participants in Finote Selam have got nutrition education during their ANC and PNC follow up. Also it is lower than a study done in Indonesia^27^. The other reasons might be due to the socio-cultural variation in educational status variation.

The present study has showed that mothers who had no education were more likely to have inadequate dietary diversity practice compared to mothers who complete more than secondary education. The present finding is supported by a research done in Angecha district^23^, and rural Indonesian^28^.

The present study has showed that employed mothers had adequate dietary diversity practice compared to house wife. This finding is supported in Aksum town^16^ and in Nepal^29^. This might be because employed mother can get money and easily afford diversified food. This finding is also supported by qualitative findings. A 28 years old lactating postpartum mother said that: *“…Since I am employed and have a monthly salary, I always buy enough amounts of food items when I receive my salary so I don’t suffer from shortage of food to my family.”*

In this study, mothers who had less than 3 meals per day were 2 more likely to had inadequate diversified diet compared to mothers who had more than 3 meals per day. This finding is supported by a study done in Debere Tabor^22^ and Nepal at pre-urban area^29^. This might be because when meal frequency increased additional food items might be added in the diet.

Lactating postpartum mothers who had time take to market more than 30 min were more likely to had inadequate dietary diversity compared to their counterpart. This result is supported by a study done in Malawi^30^. This might be because mothers who spend more time to market can face shortage of time to prepare diversified food.

Lactating postpartum mothers who didn’t get nutrition education during their ANC and PNC follow up were more likely to have inadequate dietary diversity compared to their counterpart. This finding is supported by a study done in Finote Selam^26^. This might be because mothers who get nutrition education can have more awareness about the advantage of diversified diet and can try to diversify their diet.

Mothers who had home gardening practice were less likely to have inadequate dietary diversity practice compared to their counterparts. This finding is consistent with studies conducted in Aksum, in South Gondar zone and Ataye north Shewa^16,31,32^. This might be mothers having home garden could use vegetables and fruits from their gardens for themselves and could also exchange in market by additional food items to diversify their diet.

Mothers who had big animals were less likely to have inadequate dietary diversity practice compared to mothers who had no big animals. This might be because mothers who have big animals like cows can diversify their diet by using direct products or selling milk and oxen to exchange by other food items. This finding was supported by the qualitative results. A 34-year-old lactating postpartum mother said that: *“…Since we have cows, I use milk and milk products for myself and I sell in the market to buy other food items like cabbage and oils. But I can’t sell cows for my expenditure.”*

Concerning the strength of the study, the current study was employed both quantitative and qualitative study method. Recall bias was one of the limitations of the study since some of the questions asked about the event that occurred 24 h and 4 weeks back such as 24 h dietary recall and health status. This was minimized by probing the respondents about the event. Seasonal variation was also not covered and is the other limitation of the study.

## Conclusions

The prevalence of inadequate dietary diversity practices among lactating postpartum mothers was high compared to previous studies. Having no education, employed women, meal frequency < 3 meals, time taken to market > 30 min, not received nutrition education, having a home garden, and having big animals were significant factors of inadequate dietary diversity practice. Diet habits, food taboos, low social status of women in ownership of household assets, low family support, order of feeding, child preference for resource distribution in a polygamous family, and health care provider's advice were the main mentioned reasons for inadequate dietary diversity practice. Nutrition intervention focused on nutrition education to increase meal frequency should be provided for lactating postpartum mothers to improve inadequate dietary diversity practices.

## Data Availability

The datasets used and/or analyzed during the current study are available from the corresponding author. The data will not be shared in order to preserve participant anonymity.

## Abbreviations

ANC: Antenatal care
DDS: Dietary diversity score
EDHS: Ethiopia demographic and health survey
EPI: Expanded program on immunization
FAO: Food and Agriculture Organization
GGH: Gambella general hospital
GCPH: Gambella city primary hospital
HCP: Health care provider
HEW: Health extension worker
HFIAS: Households food insecurity access scale
MDDW: Minimum dietary diversity of women
PNC: Postnatal care
SPSS: Statistical package for social sciences
WHO: World Health Organization
